# Experience of using mHealth to link village doctors with physicians: lessons from Chakaria, Bangladesh

**DOI:** 10.1186/s12911-015-0188-9

**Published:** 2015-08-05

**Authors:** Nazib Uz Zaman Khan, Sabrina Rasheed, Tamanna Sharmin, Tanvir Ahmed, Shehrin Shaila Mahmood, Fatema Khatun, SMA Hanifi, Shahidul Hoque, Mohammad Iqbal, Abbas Bhuiya

**Affiliations:** International Centre for Diarrhoeal Disease Research, Bangladesh (icddr,b), 68 Shaheed Tajuddin Ahmed Sarani, Mohakhali, Dhaka 1212 Bangladesh; School of Public Health and Community Medicine, The University of New South Wales, Kensington, NSW 2052 Australia; Institute of Development Studies (IDS), University of Sussex, Library Road, Brighton, BN1 9RE UK

## Abstract

**Background:**

Bangladesh is facing serious shortage of trained health professionals. In the pluralistic healthcare system of Bangladesh, formal health care providers constitute only 5 % of the total workforce; the rest are informal health care providers. Information Communication Technologies (ICTs) are increasingly seen as a powerful tool for linking the community with formal healthcare providers. Our study assesses an intervention that linked village doctors (a cadre of informal health care providers practising modern medicine) to formal doctors through call centres from the perspective of the village doctors who participated in the intervention.

**Methods:**

The study was conducted in Chakaria, a remote rural area in south-eastern Bangladesh during April–May 2013. Twelve village doctors were selected purposively from a pool of 55 village doctors who participated in the mobile health (mHealth) intervention. In depth interviews were conducted to collect data. The data were manually analysed using themes that emerged.

**Result:**

The village doctors talked about both business benefits (access to formal doctors, getting support for decision making, and being entitled to call trained doctors) and personal benefits (both financial and non-financial). Some of the major barriers mentioned were technical problems related to accessing the call centre, charging consultation fees, and unfamiliarity with the call centre physicians.

**Conclusion:**

Village doctors saw many benefits to having a business relationship with the trained doctors that the mHealth intervention provided. mHealth through call centres has the potential to ensure consultation services to populations through existing informal healthcare providers in settings with a shortage of qualified healthcare providers.

## Background

Bangladesh is one of 57 countries with a serious shortage of trained health human resources based on the density and quality of existing workforce [1]. According to the Bangladesh Health Watch Report 2007, there are only five qualified physicians and two nurses per 10,000 people in the country [2]. Like many countries, Bangladesh’s health care provision is pluralistic: formal health care providers co-exist with various non-formal medicinal traditions [3]. Non-formal healthcare providers (95 %) vastly out number formal providers (5 %) [4–7]. The largest group of non-formal health providers are village doctors who, in rural Bangladesh, are the first provider for 2/3^rd^ of healthcare seekers [2, 8]. Commonly reported reasons for choosing village doctors include trust in their services, ease of access, cost of treatment and positive past experience [9].

Though village doctors have little or no formal training [5, 7, 10], they practice mostly by prescribing modern medicine. In recent years, some health programs have incorporated these village doctors with variable results [11, 12]. Researchers have reported that the village doctors often prescribe unnecessary and even harmful drugs [4]. Training and regulating village doctors through social franchise resulted in improvement in their practices [13], but participating village doctors said there were financial disincentives to following the rational prescription guidelines [14]. Given the extreme shortage of trained health workers in Bangladesh and the rural population’s reliance on village doctors, it is important to try other strategies to improve the care that village doctors provide.

**Fig. 1:**
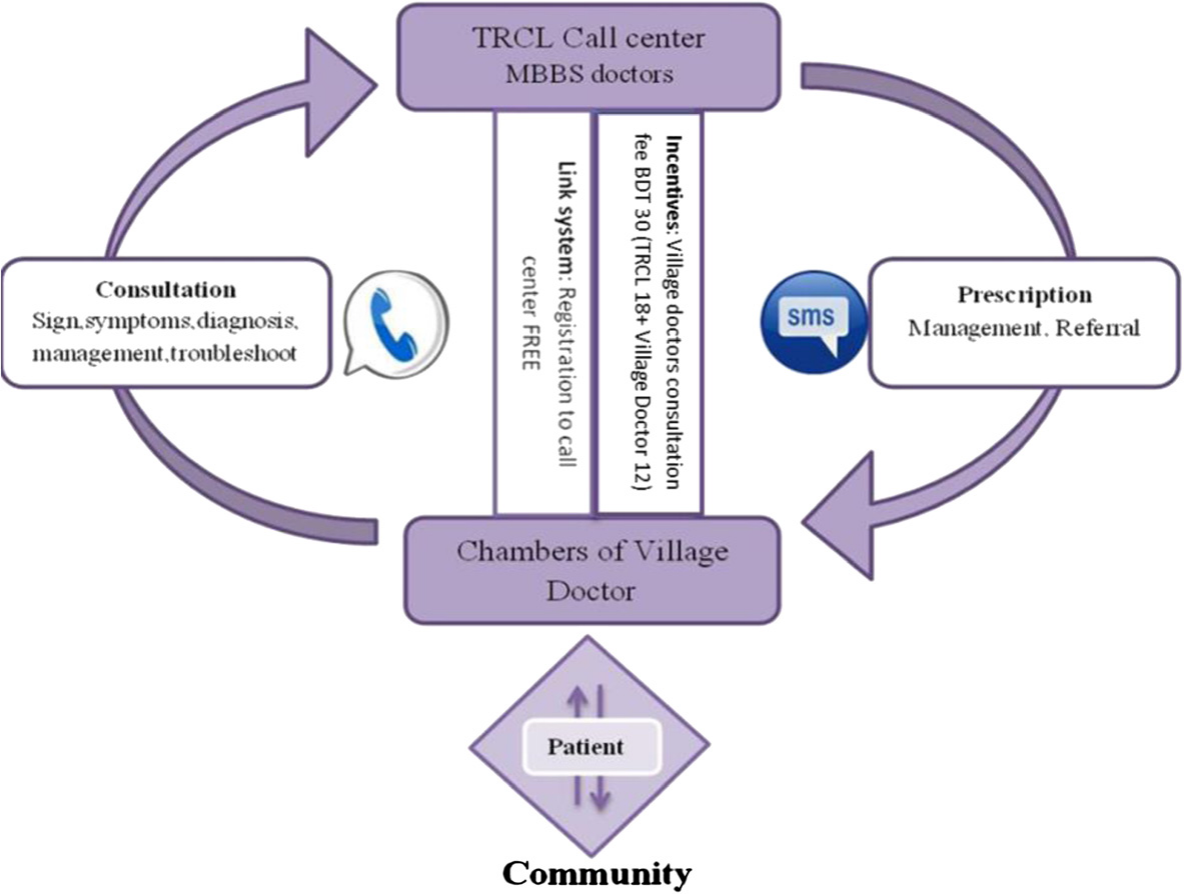
TRCL service structure

Information communication technologies (ICTs), such as mobile phones and the internet are rapidly proliferating in the developing world [15] and people are increasingly seeing ICTs as powerful tools for improving efficiency in the health sector. Mobile phones have been used to improve adherence to treatment guidelines [16] and to facilitate health workers providing care for patients [17–19]. In some programmes mobile phones have been used to increase communication both between patients and health workers, and between health workers and managers, to improve surveillance of conditions and monitoring of health products, prescriptions, and adherence to prescription guidelines [20, 21]. In Chakaria, Bangladesh, Telemedicine Reference Centre (TRCL), a private company [22] designed and implemented an mHealth intervention in 2011. The village doctors could register through a free phone call to be linked with trained physicians based at call centres. When patients sought health care from these registered village doctors, the village doctors could call the call centres for consultation. The patients were charged BDT 30 (USD 0.40) [23] for the call and the prescription from call centre was sent by SMS to the village doctor’s phone. The revenue collected from the patients was shared between the call centre and the village doctors (BDT 18 and 12 respectively) (Fig. 1). During the project period 55 village doctors were registered and 215 calls were made to the call centre [23]. The project stopped operation in early 2013 as the number of calls received from the village doctors was deemed inadequate for the business. Our study objective was to assess the mHealth intervention implemented by TRCL from the village doctors’ perspective.

## Methods

This paper presents data from Chakaria, a rural sub-district in south-eastern Bangladesh. Chakaria has similar socio demographic and health characteristics to other low performing regions in Bangladesh [4].

For our study data were collected during April to May 2013. Among the four unions where the mHealth intervention took place, three were selected considering ease of communication from the Chakaria field office of icddr,b where the researchers were based. From a pool of 55 village doctors who had participated in the mHealth intervention, 12 were selected purposefully based on their use and non-use of the mHealth intervention service.

In-depth face-to-face interviews were conducted with village doctors using a *Bangla* guideline. After preliminary analysis of the transcripts, five village doctors were re-interviewed for clarification of issues.

In the research team there were three researchers (two males and one female) trained in anthropology and experienced in qualitative data collection. The interview duration was 45 minutes to an hour and they took place at the time and place of the interviewees’ preference. Most interviews were conducted at the chamber of the village doctor during the doctor’s work hours. Field notes were also taken to record relevant information outside the scope of the interviews.

**Fig. 2:**
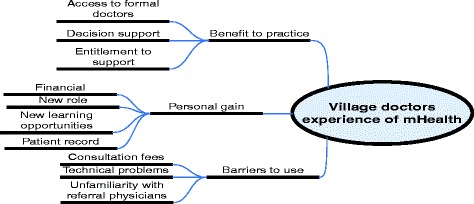
Code tree

A thematic approach was used to analyze the data [24]. The interviews were digitally recorded and transcribed for analysis. The research team reviewed the transcripts to develop a code list and transcripts were manually coded for emerging themes. The codes and subcodes were displayed in the code tree (Fig. 2). A team of researchers discussed the themes amongst themselves to come to a consensus about the codes.

Informed consent was obtained before interviewing and the study had approval from the Ethics Review Committee of icddr,b.

## Results

The respondents in our study were 30–65 years of age. They were all males. Most had at least 10 years of schooling. Five out of twelve had received government endorsed Local Medical Assistant and Family Planning training. The other seven had received short-term training from pharmaceutical companies and non-government organizations (NGOs). Most had been practicing medicine in the area for more than 20 years (Table 1).

**Table 1:** Characteristics of the respondents

Characteristics	Number *n* = 12
Schooling (years)	
1–12	9
12+	3
Training	
LMAF^a^	5
MFPC^b^	1
Village Doctor training	3
VHWTC^c^	1
Community Paramedics	2
Duration of training (months)	
Mean	15
Range	4–36
Duration of practice (years)	
1–10	4
21–30	5
31–40	3

^a^Local Medical Assistant and Family Welfare

^c^Village health worker training course

^b^Medical Faculty of *Polli Chikitshok*

The respondents described various benefits and challenges around using the mHealth program. Their experience revolved around benefits to their practice, personal benefits, and barriers they faced in using mHealth.

### Benefits to their practice

The village doctors perceived that their practice was benefited from participating in the mHealth project in three different ways: access to formal doctors, decision support, and the ability to provide services at any time.

#### Access to formal doctors

The TRCL scheme offered the village doctors remote access to qualified doctors. Village doctors described several advantages of the scheme: they appreciated having access to qualified doctors; they talked about it being easy for them to call the call centres and receive prescriptions; they said that the SMS-based prescriptions were accessible and that they could follow the procedures without problems; they spoke about the scheme improving their own service quality; and they were hopeful that adding the program to their existing practice would attract additional business. As one village doctor said:

*It’s not only about my income, if a patient doesn’t get proper treatment from me and they don’t recover then they will not come to me next time and in that case how will I earn in future* [sic]*? So with the help of TRCL we are able to provide better treatment and ensure that more patients come.* IDI_6

#### Decision support

The village doctors felt that the support of the call centre allowed them to increase the breadth of their services. Previously, the responding village doctors had referred many patients to other medical practitioners because they could not decide on diagnoses and treatments. With the help of TRCL, village doctors felt better equipped to deal with difficult health problems. One respondent mentioned,

*You know I provide treatment with the help of TRCL, one patient came with arthritis and one small child with a urine problem….. I prescribed antibiotic but it wasn’t working. I called TRCL service and with help of them I was able to treat those patients.* [sic] IDI_3

Another village doctor talked about referring patients to hospitals more appropriately after enrolling in the TRCL program:

*I referred a few patients after registering with this project. In the beginning we used to not refer any patient but after a few months I referred some (patients). Normally I referred poor patients to the government hospitals. I referred one asthma patient to the Chittagong hospital after consulting over phone [TRCL], this was a very critical patient and TRCI doctor suggested not to send him (patient) to any sub-district or district level hospital. I followed his suggestion and saved time.* [sic] IDI_2

#### Entitlement to support

The formal affiliation with TRCL allowed the village doctors to feel comfortable about calling the qualified doctor for advice. Consulting with qualified doctors was not a new experience for the village doctors as many worked as assistants to qualified doctors or had personal links to them. They consulted these doctors when they felt that a patient’s situation was beyond their experience. However, these links were informal and based on personal relationships and the village doctors often felt hesitant to call the qualified doctors outside of normal office hours and there was no assurance that a qualified doctor would be available when needed. In contrast, being part of the TRCL entitled the village doctors to call qualified doctors at any time of the day or night for assistance. Paying for the services of the qualified doctors was key for creating the sense of entitlement. One village doctor said:

*Though the X [name of the qualified doctor] doctor did not mind if I call him for help, I have felt shy about calling him (in an odd time). I always used to worry (about what he would think). Sometimes I called him anyway because I have no other way. But I didn’t feel shy about calling the TRCL doctor because I am paying; it may be 1 Taka (BDT) still I am paying something for the services*. [sic] IDI_7

### Personal gains

During in-depth interviews village doctors described their perceptions of both financial and non-financial benefits of the TRCL program and the potential for such programs in the future. All the village doctors interviewed were drug dispensers and depended on the money from drug sales for their livelihood. In normal practice they did not charge for consultation.

#### Financial

During the TRCL program the village doctors were allowed to charge their clients 30 taka/call (US$0.40) to the TRCL call centre. The village doctors were given a share of this consultation fee which added to their earnings; they also sold and dispensed the medication prescribed by the qualified doctors which made them benefit from drug sales. The village doctors found that the TRCL system was an inexpensive option for giving the community access to qualified doctors, as by comparison, the fee for face-to-face consultation with a qualified doctor was 200–300 taka (US$2.5–3.75). In addition many of the qualified doctors lived in the urban centres and people would have to incur time and transportation costs to avail qualified doctors’ services. Village doctors felt they were providing the community a convenient and inexpensive option for medical consultation with a qualified doctor and that many people were willing to pay for it. The village doctors also felt that the official link to the qualified doctor enhanced their reputations and increased their patient flow. Credibility in the community was a very important element of the village doctors’ business. As one village doctor stated:

*One of my patients asked me to call the qualified doctor (for his health problem). The doctor provided a prescription over phone and I provided the medicine accordingly. The patient was very impressed as he received treatment from a qualified doctor for only 30 taka.* [sic] IDI_3

#### New learning opportunities

During the interviews, four village doctors mentioned that their own clinical, diagnostic, and disease management skills improved through the TRCL program. In the calls to a TRCL doctor, the village doctors received prescription and case management advice, opening new learning opportunities. The project also gave them experience in dealing with critical conditions. According to one village doctor:

*This is a great benefit for us, we become experienced to treat some new illness, and we get confidence to serve the ill people. At the same time people also have showed their trust in us…… previously we were not confident in giving treatment but when they (TRCL doctors) provide direct assistance, we feel confident to treat.* [sic] IDI_4

#### New role

The village doctors who used the TRCL program saw a special role for themselves in the formal health care system. They talked about villagers having difficulty explaining their health problems to the qualified doctors. Communication problems were exacerbated in Chakaria because the local dialect is not easily understood by people from other parts of Bangladesh. (The communication issues were a barrier for the village doctors as well, described later in detail). Village doctors in Chakaria- conversant both in the local dialect and in medical terminology–saw themselves as a conduit between the community and the qualified doctor. As one village doctor stated:

*It’s always good to communicate with the doctor on (one’s) own but if it is not possible due to dialect (differences), you cannot make the doctor understand your problem. In such case, if local village doctor communicates on (patient’s) behalf, it makes the care seeking more convenient.* [sic] IDI_10

#### Patient record

Patient records were another benefit of the TRCL program as a record of medical history and general information was created for any patient who called in. The village doctors found these records useful for providing a continuum of care. As one village doctor said,

*During service provision we write down the main illness problem and keep it. It will help us for the follow up. When the patient comes for follow up, we can easily see the previous prescription and find the problem within short time. It was an easy procedure to give accurate treatment*. [sic] IDI_12

In addition, any prescription sent by text message to the village doctor had the name of the medicine, dosage, and related advice. The village doctors would provide this information to patients on handwritten prescriptions and could save the information for future use. This record keeping system helped the village doctors provide accurate treatment and enhanced their prestige.

The village doctors talked about people liking the short message services (SMS) prescription better than the verbal prescriptions provided by other village doctors who were not in the program as they felt that they were receiving better quality service. One village doctors described a situation with a client in the following manner:

*One of my patients told me that, “most of the village doctors always prescribed us medicine verbally and they never give any written prescription. So every time when I need to buy medicine I had to visit this village doctor to get the medicine name because I don’t know the medicine name and don’t have the written prescription. But you [registered* village doctors*] are giving us written prescriptions so now I can buy medicine from any medicine shop even though I am away from this area. I think if we kept this written prescription we need not to visit you again so this will save our time and ensure accurate care.* [sic] IDI_11

To summarize, the village doctors identified a range of ways in which they from the intervention benefitted, including increased status and prestige, new mechanisms to improve treatment (through SMS prescriptions and patient records), financial remuneration, increased medical skill, and improved diagnosis capability. The intervention was not, however, without problems.

### Barriers to use

When village doctors used the call centre services they experienced some barriers which were related to charging consultation fees, facing technical problems, and trusting the unfamiliar doctors in the call centre.

#### Consultation fees

Some village doctors pointed out that their clients were not expecting to pay for consultations and, therefore, were not ready to pay. It was important for the community to be made aware of the importance of the enhanced services provided by the village doctors participating in the TRCL intervention. When people were not aware that the village doctors were linked with trained doctors and could not see the trained doctor in person, they were not willing to pay the consultation fee. As one village doctor said,

*Usually people don’t pay our fees. They only pay for the medicine. Now when you ask 30 taka for consultation fees then they have doubt about this. They don’t know whom I was talking to so there is a chance of mistrust*. [sic] IDI_6

Furthermore, among the clientele of the village doctors free consultation was considered an important benefit. To avail the TRCL intervention a client had to pay cash or the village doctors had to provide credit which was seen as a problem. As one village doctor said,

*Sometimes I have to pay for my patient, so* say if *I have to pay taka 10 per patient, then for 10 people it will be 100 taka. Surely I will be broke if I go on paying like this.* [sic] IDI-1

#### Technical problems

During the interviews every respondent mentioned having technical problems with the calls. They found that call drop, call waiting, difficulty navigating the automated system, and delay in receiving the SMS prescriptions made using the call centre difficult at times. Long waits to connect to the system or receive prescriptions increased waiting time for their clients and the village doctors found delays inconveniencing their clients unacceptable. As one village doctor said:

*Sometimes when we call to the call centre’s doctor for consultation and can't connect with their phone, we try again and again. And then sometimes some patients become annoyed. And many times they didn’t want to wait. They wanted to visit another doctor [MBBS], they said why they would wait here? Better they can go to the Upazilla headquarters to see a ‘Boro’ (qualified) doctor.* [sic] IDI_7

Five of 12 village doctors mentioned that they eventually stopped calling the call centre because of these technical problems. TRCL was notified of the technical problems, but the fixes were not necessarily timely; further, when the program did not generate adequate revenue TRCL stopped being responsive to the village doctors.

#### Unfamiliarity with referral physicians

All of the village doctors are local residents with strong community ties. Their relationship with the community is an important piece of capital for their business. By contrast, all the TRCL doctors were based in distal urban areas and were not familiar with the language and culture of the area. The disjunction with the TRCL doctors left the village doctors feeling constrained about communicating in their local dialect and not fully comfortable talking with the consulting doctors. Some village doctors mentioned difficulty in describing patients’ conditions in *Bangla* and the TRCL doctors found it difficult to understand the village doctors’ dialect. Two of the village doctors had problems speaking and understanding *Bangla* adequately. One village doctor mentioned,

*(Some) people from this area only speak in local dialect. When they call to the call centre’s doctor, then (the doctor) does not understand the local language. This is a problem.* [sic] IDI_10

For the village doctors there was also a feeling of disparity in professional stature and education between themselves and the call centre doctors that impeded the building of a good working relationship. Two village doctors reported that the lack of comfort with the call centre doctors resulted in their reluctance to use the intervention. As one village doctor said,

*When I talk with my patient I feel like we are brothers during our conversation and the patient feels comfortable (about sharing problems). But when I consult with call centre sometimes I didn’t feel that warmth probably because they are from another place and we never met.* [sic] IDI 10

To summarize, from the village doctors' perspective, charging consultation fees, technical problems, and lack of familiarity between qualified doctors and the village doctors were barriers to the use of the intervention.

### Suggestions for improvements of the mHealth project by village doctors

As the village doctors saw many potential personal and business benefits from the intervention, they suggested specific improvements that would make it more acceptable and useable for them.

#### Advertising to create awareness

The intervention was not advertised adequately and people in the community did not know about it. The village doctors said that if the community knew about the enhanced services that registered village doctors were providing, trust would be built and people would be willing to pay the consultation fee. The village doctors suggested that advertisement campaign through village fairs could promote the service.

#### Solving the technical problems

The village doctors wanted the technical network problems solved to improve their ability to access the call centre with minimal delay and charge. They also emphasized the importance of the timely arrival of the SMS prescriptions for the system to gain popularity.

#### Provision for the poor

Village doctors’ existing services are designed with flexible payment schemes that allow poor people access healthcare and they suggested that a special scheme could be designed for people who could not pay the consultation fee.

## Discussion

Health care in Bangladesh, as in much of the developing world, is in crisis. Much of the crisis rests on the serious scarcity of trained medical professionals which deprives millions of life-saving health services. Two general ideas have been tried to stretch health services to those in need: one, task shifting where lay health workers and village doctors provide health care in place of trained medical professionals [3, 11], and two, the flourishing e and mHealth sector where trained medical professionals deliver health services remotely. In the context of Bangladesh, our study is highly interesting and provocative on at least two fronts: 1) combining task shifting with e and mHealth for greater reach of competent care, and, most crucially, 2) village doctors–a group generally found intractable in their problematic practices–engaged in ways that has potential to improve their practices.

Village doctors and other informal health care providers are the main source of health care available to the poor in Bangladesh [3–5, 25, 26] and in other developing countries [5, 27]. So, even without purposeful task shifting, much health care is falling outside of the formal health care system and its trained health care providers. Though village doctors have successful business practices, they and other informal health care providers in Bangladesh and elsewhere are inadequately trained [5, 28], with the training they have coming from unregulated private institutions [29]. Their medical practices, particularly their provision of drugs, are often irrational or harmful [28, 30]. And previous studies and pilot programs to provide training to improve village doctors’ practices have failed to show adequate results [13] because following the training and improving their drug selling practices would unacceptably diminish their income. Critically and in contrast, in our study we found that village doctors saw benefits in being linked to the formal health care system, were desirous of the benefits from those links, and invested in creating and fostering links with qualified doctors. And, further, by providing financial incentives for desired outcome, the links could potentially improve the worst of the village doctor practices–the inappropriate prescribing of modern medicines.

Though the village doctors engaged with the TRCL program, they faced various technical difficulties that diminished their experience of using the call centre. Network interruptions, call drop, navigation of the system to reach the call centre doctor, and delays in receiving prescription all deterred village doctors from using the intervention. It is likely that these technical problems were at least partially responsible for the low knowledge and use of the mHealth program services in similar community [31]. Such lack of knowledge about the existence of programs is not uncommon and the lack of adequate infrastructure and systems has been recognised as an important barrier to the proliferation and use of ICTs in developing countries, including Bangladesh [32–35]. Village doctors’ relatively poor education and familiarity with technology probably exacerbated the technical problems they encountered and reported; other researchers working in low literacy populations have reported finding similar problems in the accessing of technology-based services [22, 36]. This finding points to the special need for capacity building for users of technology-based systems in the future.

Additionally, socio-cultural difficulties including language barrier, power differentials, and lack of comfort with the call centre doctors diminished the use of the system. In the trust literature, researchers have found that people have more trust in personal relationships and personal experiences with a service provider and less trust in sources of information with which they have no personal experience [37]. The village doctors did not have personal relationship with the call centre doctors. The vast differences in education, language, and expertise between village doctors and the qualified doctors also created a sense of discomfort, which was also reported in another study where community health workers were linked to qualified doctors [35]. Some of the lack of comfort with call centre doctors could have been due to village doctors’ quality perceptions related to remote diagnoses where the village doctors felt a language barrier. In a Bangladesh study of toll free mobile communication between mothers and community health workers for obstetric complications, both community health workers and mothers expressed discomfort regarding the quality of the remote diagnoses [32]. Other researchers have reported that user perceptions of quality of service is a strong determinant of the use of e and mHealth platforms [38]. Substantial effort is needed to create a better relationship between the informal and formal health care providers in the future for similar projects to be effectively delivered.

A pillar of Bangladesh’s health achievements has been the availability of low-cost, life-saving drugs [3, 39, 40]. A progressive National Drug Policy has led to a surge of national pharmaceutical companies. Through village doctors and other informal health care providers these pharmaceutical companies distribute drugs to communities throughout Bangladesh [30]. It is not realistic to expect a telemedicine service linking village doctors to qualified doctors to curb irrational drug prescriptions without significant efforts at enforcing laws linking training and the legal permission to prescribe. However, it is important to find innovative ways to bring village doctors under some regulatory mechanism so that their practices improve: mobile technology could be used to create such a mechanism.

The strength of our study was that it provided insights about linking informal health care providers with qualified doctors through call centres. The study allows us to understand the perspectives of the village doctors -an important link between rural communities and the formal health sector.

Our study had a few important limitations. We did not evaluate the impact of the intervention from the call centre provider end. Although we know the number of calls and reasons for which they were made by the village doctors, we don’t know how many calls were not successfully registered due to technical problems. We did not explore why the technical problems were not solved and why the service was discontinued beyond that it was not seen as a feasible business for the provider. In addition, since the project was stopped in early 2013, we do not have data on its impact on the community’s health and village doctor behaviour.

## Conclusion

Village doctors found that being linked with qualified doctors through call centres improved both their personal capacity and their business. However, trust building exercises between themselves and qualified doctors would benefit both their relationship with qualified doctors and programs like TRCL. In addition technical barriers need to be addressed for similar future programs to be useful to the village doctors. Due to the serious lack of trained health workforce, village doctors are an important part of healthcare provision in Bangladesh and they can act as a complement to the formal health system. It is important to assess the impact of similar mHealth interventions on village doctors’ treatment patterns for such interventions to contribute to the health goals of the country.

## Abbreviations

BDT: Bangladeshi Taka
ICT: Information Communications’ Technologies
IDI: In depth Interview
mHealth: Mobile Heath
NGO: Non Government Organization
SMS: Short Messages Service
TRCL: Telemedicine Reference Centre
USD: US dollar
